# A Preferentially Segregated Recycling Vesicle Pool of Limited Size Supports Neurotransmission in Native Central Synapses

**DOI:** 10.1016/j.neuron.2012.08.042

**Published:** 2012-11-08

**Authors:** Vincenzo Marra, Jemima J. Burden, Julian R. Thorpe, Ikuko T. Smith, Spencer L. Smith, Michael Häusser, Tiago Branco, Kevin Staras

**Affiliations:** 1 School of Life Sciences, University of Sussex, Brighton BN1 9QG, UK; 2 Medical Research Council Laboratory for Molecular Cell Biology and Cell Biology Unit, University College London, London WC1E 6BT, UK; 3 Wolfson Institute for Biomedical Research, University College London, London WC1E 6BT, UK; 4 Department of Neuroscience, Physiology, and Pharmacology, University College London, London WC1E 6BT, UK; 5 Neuroscience Center, University of North Carolina School of Medicine, Chapel Hill, NC 27599, USA; 6 Department of Cell Biology and Physiology, University of North Carolina School of Medicine, Chapel Hill, NC 27599, USA

## Abstract

At small central synapses, efficient turnover of vesicles is crucial for stimulus-driven transmission, but how the structure of this recycling pool relates to its functional role remains unclear. Here we characterize the organizational principles of functional vesicles at native hippocampal synapses with nanoscale resolution using fluorescent dye labeling and electron microscopy. We show that the recycling pool broadly scales with the magnitude of the total vesicle pool, but its average size is small (∼45 vesicles), highly variable, and regulated by CDK5/calcineurin activity. Spatial analysis demonstrates that recycling vesicles are preferentially arranged near the active zone and this segregation is abolished by actin stabilization, slowing the rate of activity-driven exocytosis. Our approach reveals a similarly biased recycling pool distribution at synapses in visual cortex activated by sensory stimulation in vivo. We suggest that in small native central synapses, efficient release of a limited pool of vesicles relies on their favored spatial positioning within the terminal.

## Highlights

► Native hippocampal synapses have a small spatially biased recycling vesicle pool ► Pool size is regulated and positioning near the active zone relies on actin turnover ► In vivo sensory-activated synapses in visual cortex share same organization ► Positional bias ensures effective transmission in central size-limited terminals

## Teaser

Using a functional-ultrastructural approach, Marra et al. demonstrate that recycling vesicles in native synapses form a small subset of the total vesicle population. The spatial bias of this functional pool toward the release site helps to support efficient neurotransmission.

## Introduction

Most information transfer in the CNS depends on fast transmission at chemical synapses, and the mechanisms underlying this process have been extensively examined. In particular, much attention has focused on presynaptic terminals, characterized by their cluster of neurotransmitter-filled vesicles lying close to a specialized release site (Siksou et al., 2011). Although synaptic vesicles appear morphologically similar, they are, in fact, organized into functionally discrete subpools that are key determinants of synaptic performance (Denker and Rizzoli, 2010; Rizzoli and Betz, 2005; Sudhof, 2004). Understanding the specific relationship between these functional pools and their organizational and structural properties is thus a fundamental issue in neuroscience. Specifically, several key questions merit attention. What is the absolute size of the functional vesicle pool at a synapse and how does its magnitude relate to other parameters of the synaptic architecture? Do functionally distinct subpools have a specific spatial organization that reflects or supports their operational roles? If so, what molecular substrates regulate this organization and what are the consequences for synaptic function?

Addressing such questions is challenging because it requires a readout of functional synaptic vesicle pools that can be identified in ultrastructure (de Lange et al., 2003; Denker et al., 2009, 2011; Harata et al., 2001b; Henkel et al., 1996; Paillart et al., 2003; Richards et al., 2000, 2003; Rizzoli and Betz, 2004; Schikorski and Stevens, 2001; Teng and Wilkinson, 2000). This challenge is particularly acute when considering native synapses within their specific cytoarchitecture. The most informative results to date have come from studies of large and mainly peripheral synapses, from which a consensus has emerged regarding vesicle structure-function relationships. At the frog neuromuscular junction, terminals contain substantial populations of vesicles organized into functional subpools (Rizzoli and Betz, 2005); elegant ultrastructural evidence has shown that the vesicles belonging to the readily releasable pool comprise a small subset (∼15%–20%) (Richards et al., 2000, 2003; Rizzoli and Betz, 2004) of the total vesicle population and are randomly spatially distributed within the terminal (Rizzoli and Betz, 2004). A similar lack of spatial segregation has been shown in *Drosophila* neuromuscular junction (Denker et al., 2009), the mammalian calyx of Held (de Lange et al., 2003), and isolated retinal bipolar nerve terminals (Paillart et al., 2003). Thus, in these large multirelease site synaptic junctions, the spatial positioning of recycling vesicles appears to be largely irrelevant for functional vesicle properties (Denker et al., 2009).

**Figure 1:**
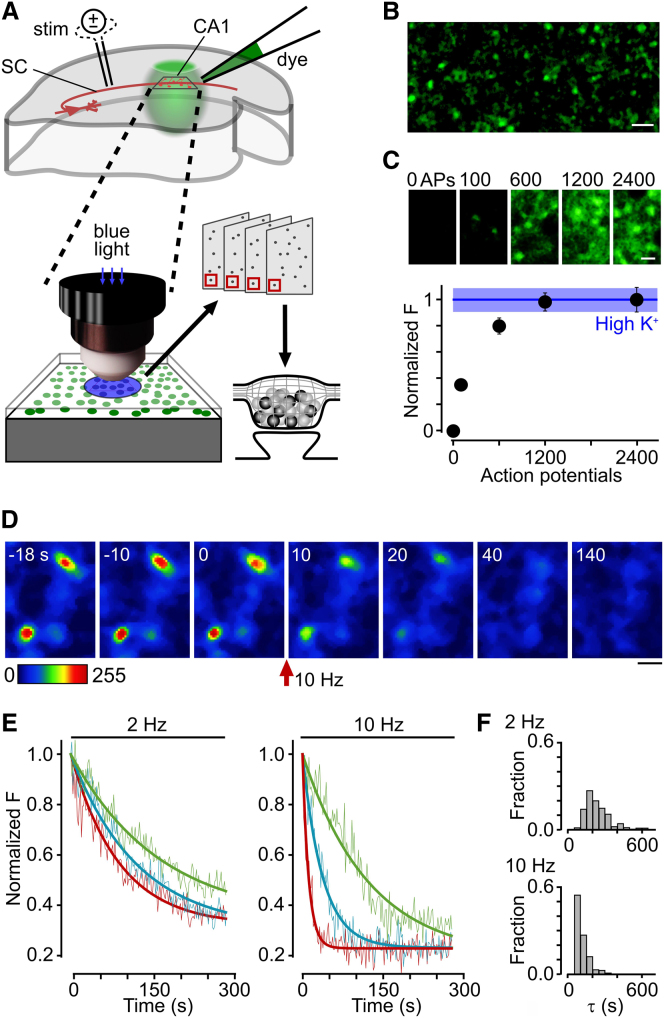
Heterogenous Release Properties of Functional Vesicle Pools in Acute Hippocampal Slice (A) Schematic illustrating experimental protocol for visualizing recycling vesicles at fluorescence and electron microscope level using photoconversion of FM1-43FX. SC, Schaffer collateral fiber. (B) Typical sample image of FM dye-positive fluorescent puncta in CA1, labeled using a 10 Hz 1,200 AP loading stimulus. Scale bar represents 5 μm. (C) Dependence of FM dye-labeling intensity at synaptic terminals using a range of different loading protocols at 10 Hz (0 APs, 100 APs, 600 APs, 1,200 APs, and 2 × 1,200 APs). Top: typical sample images for each loading condition. Scale bar represents 1 μm. Bottom: plot showing average labeling intensity of fluorescent puncta for each loading condition normalized to intensity with 40 mM KCl (line and blue band show mean ± SEM). Labeling intensity saturates at 1,200 APs (Kruskal-Wallis test, p < 0.0001; Dunn’s test shows p values < 0.0001 for the following groups: 0 AP versus 600 AP, 0 AP versus 1,200 AP, 0 AP versus 2,400 AP, 0 AP versus High K^+^, 100 AP versus 600 AP, 100 AP versus 1,200 AP, 100 AP versus 2,400 AP, and 100 AP versus High K^+^, all other comparisons between groups were nonsignificant, p > 0.05). Plot shows mean ± SEM. (D) Sample time-lapse images demonstrating stimulus-driven dye loss at synaptic puncta (10 Hz). Scale bar represents 1 μm. (E) Sample traces with fitted exponential curves illustrating heterogeneity of destaining rates for 2 and 10 Hz stimulation. (F) Frequency histograms of destaining time constants for 2 Hz (n = 141 synapses) and 10 Hz (n = 156 synapses).

How do these findings relate to functional vesicle pools in small native central synapses? So far, such studies have been almost exclusively limited to cultured neurons (Harata et al., 2001b; Schikorski and Stevens, 2001), but the relevance of these observations for native synapses remains unknown. Here we used an approach based on stimulus-driven fluorescence labeling of recycling synaptic vesicles, dye photoconversion, and serial section electron microscopy in acute hippocampal brain slices and visual cortex in vivo to address these questions (Figure 1A). This method allows us to make comparisons between the functional recycling pool and other ultrastructural parameters within the same terminals. In hippocampal synapses, we demonstrate that the functionally recycling vesicle fraction is, on average, only a small subset (approximately one-fifth) of the total pool, is highly variable across the synaptic population, and is regulated by cyclin-dependent kinase 5 (CDK5) and calcineurin activity. Spatial and cluster analyses reveal a clear positional bias in the presynaptic vesicle cluster where recycling vesicles tend to occupy sites nearer to the active zone. Actin remodeling contributes to this spatial segregation and filament stabilization perturbs vesicle release properties, suggesting that vesicle positioning has functional consequences for signaling efficacy. Experiments in visual cortex in vivo, in which functional vesicles are dye labeled by visually driven activity, reveal a similar spatial organization, supporting the idea that this is a conserved feature across different types of small central synapse. Our findings suggest that a small recycling pool supports neurotransmission in native central synapses and that the physical position of recycling vesicles in the terminal is an important factor in their favored stimulus-driven fusion.

## Results

### Vesicle Recycling Properties in Native Tissue

To label functional vesicle pools in native hippocampal tissue, we prepared acute slices from rat brain and activated CA3 axons while the styryl dye FM1-43 (Betz and Bewick, 1992; Gaffield and Betz, 2006; Ryan et al., 1993) was applied to a target region in CA1 (Zakharenko et al., 2001) (Figure 1A). Confocal imaging revealed clear punctate fluorescent staining (Figure 1B), the intensity of which was stimulus dependent (0–2,400 action potentials [APs]), consistent with the loading of synaptic vesicles in presynaptic terminals (Figure 1C). Labeling intensity reached saturation when electrical stimulation exceeded 600 APs and this maximal load was not significantly different from the intensity of synapses labeled with hyperkalemic stimulation (Figure 1C, bottom, see figure legend for statistics).

Next, we tested whether labeled terminals were release competent by monitoring fluorescence intensity during a further round of stimulation. Synapses readily underwent activity-evoked destaining consistent with exocytosis and dye loss (Figures 1D and 1E). Across the synaptic population, the timecourse of destaining became faster as the stimulation frequency increased but was highly variable between terminals (Figures 1E and 1F), reflecting substantial heterogeneity in individual synaptic release properties similar to previous findings in cultured hippocampal neurons (Branco et al., 2008; Murthy et al., 1997; Waters and Smith, 2002; Welzel et al., 2011). To establish that the recycling pool accessed during these destaining experiments had the same composition as the pool that was dye marked during the loading protocol—in other words that it was preferentially reused—we compared our experimental dye loss profiles to simulated destaining curves based on the reuse of varying fractions (0%–100%) of the recycling pool (see Experimental Procedures). The experimental data were best described by the simulated destaining profile corresponding to ∼90% vesicle reuse (see Experimental Procedures), implying that the recycling pool was essentially immutable over the timecourse of our experiments. These results demonstrate the robust stimulus-driven FM dye labeling and subsequent reuse of functionally recycling synaptic vesicles in native hippocampal slice.

### Ultrastructural Characterization of the Functional Vesicle Pool

**Figure 2:**
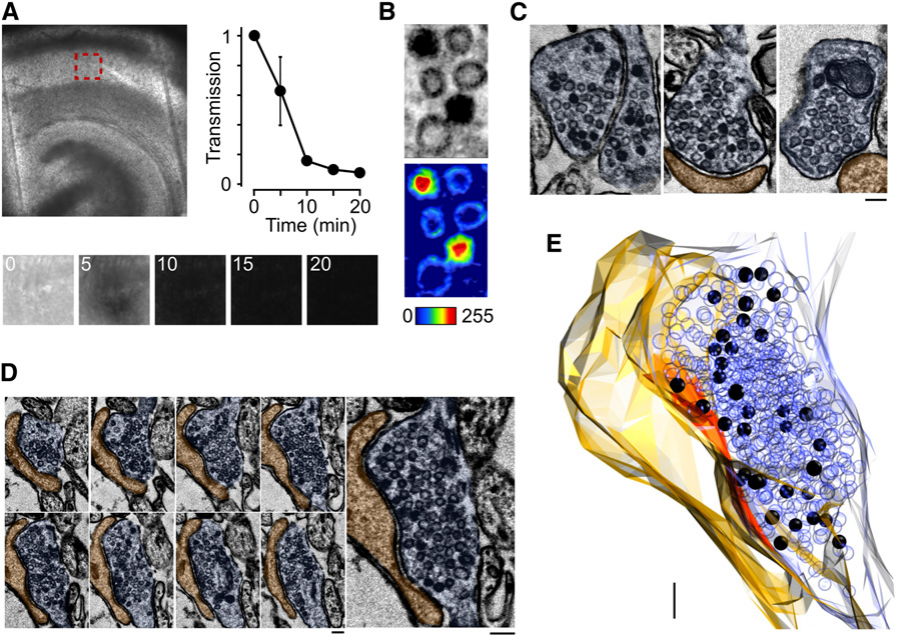
Ultrastructural Visualization of the Functional Vesicle Pool in Native Synapses (A) Photoconversion of target region in CA1. Top: bright-field image of fixed hippocampal slice before photoconversion with red box indicating target region. Bottom: photoconversion reaction in target region for 0, 5, 10, 15, and 20 min time points. Right: mean ± SEM line plot showing reduced bright-field light transmission with progression through photoconversion reaction. (B) Top: electron micrograph showing the different appearance of PC+ and PC− vesicles. Bottom: intensity plot with pseudocolor look-up table illustrates different lumenal density profiles for PC+ (red lumen) and PC− (blue lumen) vesicles. (C) Left and middle: examples of synapses with PC+ and PC− vesicles. Right: example synapse from control slice for which no stimulation was delivered during the FM dye-labeling protocol (see Figure S1). (D) Consecutive serial electron micrographs of a synapse containing PC+ vesicles. (E) Full three-dimensional reconstruction of the same presynaptic terminal from (D) based on 13 consecutive serial sections. PC+ vesicles appear as black spheres and PC− as empty spheres with active zone in red. Scale bars represent 100 nm.

Next, we used an experimental approach that allows dye-labeled functional vesicle pools to be visualized at ultrastructural level. We took advantage of the fact that vesicles labeled with FM dye can readily photoconvert diaminobenzidine (DAB) to an osmiophilic polymer (Darcy et al., 2006a; de Lange et al., 2003; Denker et al., 2009, 2011; Henkel et al., 1996; Rizzoli and Betz, 2004; Schikorski and Stevens, 2001; Teng and Wilkinson, 2000). In this way, recycling vesicles can be discriminated from nonrecycling vesicles in electron micrographs by their increased vesicle lumen opacity. Loaded slices were rapidly fixed, incubated in DAB, and bubbled with oxygen before photoillumination with wide-field epifluorescence to drive photoconversion. Calibration of the illumination time needed to yield a maximal photoconversion product was established by monitoring light transmission through the tissue (Figure 2A). Target regions of the slice were then processed, embedded, and sectioned for visualization in the electron microscope.

**Figure 3:**
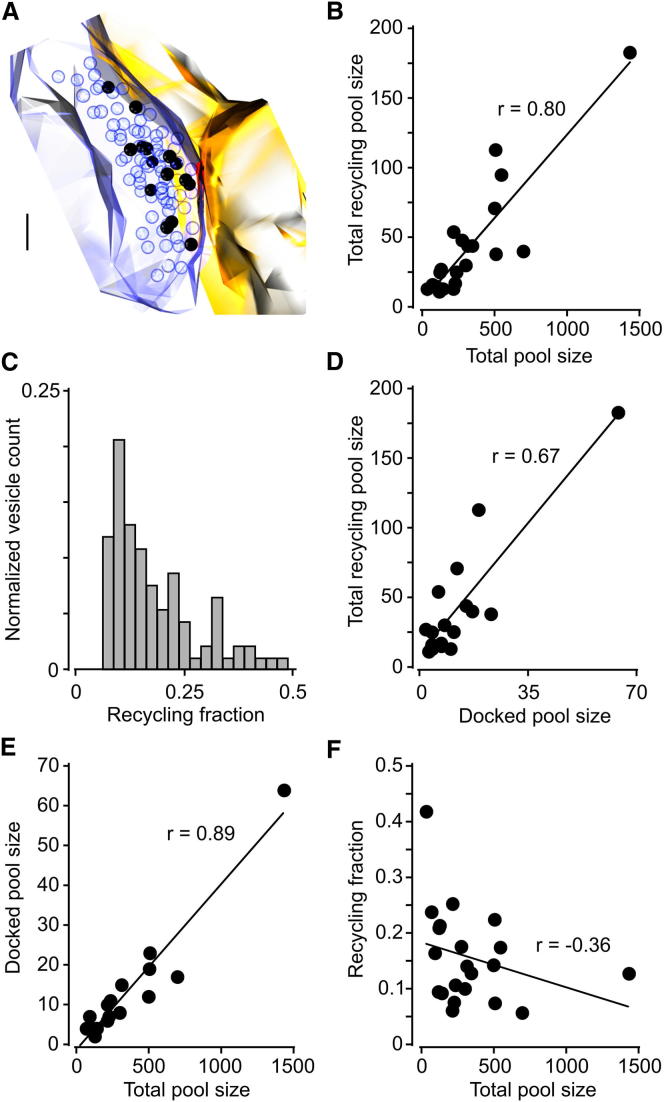
Quantification of the Functional Vesicle Pool in Native Synapses (A) Full three-dimensional reconstruction of sample synapse used for quantification. Scale bar represents 100 nm. (B) Scatter plot and regression line for total recycling pool size versus total pool size for 21 fully reconstructed synapses (two-tailed Spearman’s test, r = 0.80, p < 0.0001, n = 21). (C) Frequency histogram for recycling pool fraction for 93 synapses, illustrating a broad spread in the relative magnitude of the recycling pool. (D–F) Scatter plot and regression lines for comparisons between synaptic parameters for serial section reconstructions. (D) Total docked vesicles versus total pool size (two-tailed Spearman’s test, r = 0.67, p < 0.05, n = 17). (E) Total recycling pool size versus total docked vesicle number (two-tailed Spearman’s test, r = 0.89, p < 0.0001, n = 17). (F) Fractional recycling pool size versus total pool size (two-tailed Spearman’s test, r = −0.36, p = 0.11, n = 21).

At ultrastructural level, FM dye-labeled slices were characterized by synapses containing photoconverted (PC+) and nonphotoconverted (PC−) vesicles (Figures 2B and 2C). In control experiments, we confirmed that the number of PC+ vesicles was negligible when slices were not stimulated during the labeling protocol and zero without photoillumination (see Figure S1 available online). To measure the size of the recycling vesicle pool, we examined full serial reconstructions from maximally loaded synapses and counted the total number of PC+ vesicles (Figures 2D, 2E, and 3A, see Experimental Procedures). This yielded an average recycling pool size of 45 ± 9 vesicles, a small proportion of the total vesicle pool (331 ± 67 vesicles, n = 21 reconstructed synapses). Notably, however, the number of recycling vesicles was highly variable across the synaptic population, illustrated by a high coefficient of variation (0.94).

To address what might underlie this variability, we compared our ultrastructural readout of the functional pool against other morphological parameters from the same terminals (Harris and Sultan, 1995; Murthy et al., 1997, 2001; Schikorski and Stevens, 1997, 2001). First, we examined how the absolute size of the recycling pool relates to the total vesicle population. This revealed a strong positive correlation (Figure 3B), but the plot was characterized by a broad scatter around the regression line, suggesting that the fraction of total vesicles that recycle was highly variable (Figure 3C). A similar relationship was observed when the recycling pool was plotted against the number of vesicles docked at the active zone (Figure 3D), another parameter that scales with the total pool (Figure 3E). Notably, the recycling vesicle fraction showed no correlation with the total pool size (Figure 3F). Thus, in native tissue the maximal available recycling pool is highly variable but, on average, represents a small fractional subset of the total pool (0.17 ± 0.01, n = 93). This variability is a consequence of a broad spread in recycling fractions that is independent of the total vesicle number, suggesting that factors other than the absolute size of the synapse are important in regulating the functional pool fraction. To explore neuronal activity as a potential factor influencing the recycling pool fraction, we also carried out experiments using a lower frequency loading protocol (1,200 APs, 4 Hz). We found that the mean recycling fraction (0.17 ± 0.01, n = 68) was essentially identical to the 10 Hz loading condition (p = 0.52, two-tailed Mann-Whitney test) and similarly variable (Figure S2), suggesting that stimulus frequency was not a critical determinant of the functionally recruited pool size.

### Preferential Proximity of Functional Vesicles to the Active Zone

**Figure 4:**
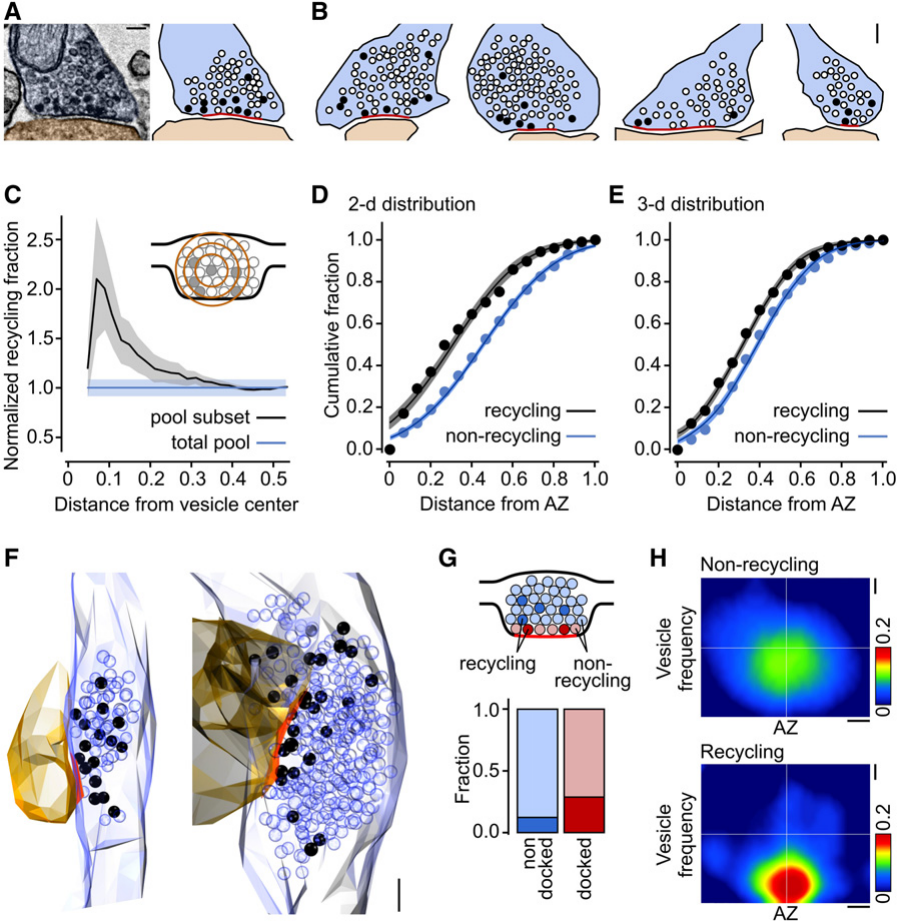
Preferential Distribution of the Recycling Vesicle Pool toward the Active Zone (A) Sample electron micrograph and corresponding cartoon illustrating the organization of recycling vesicles (black circles) and nonrecycling vesicles (open circles) in relation to the active zone (red). Scale bar represents 100 nm. (B) Sample cartoons showing vesicle positions for four other synapses. Scale bar represents 100 nm. (C) Cluster analysis plot showing the mean recycling vesicle fraction for the vesicle population surrounding PC+ vesicles with increasing distance from the vesicle center (see inset). (D) Summary cumulative frequency plot of linear distances from vesicles to active zone for recycling and nonrecycling vesicles from 24 central synaptic sections. Line and shading indicate data fits and 95% confidence intervals. (E) Summary cumulative frequency plot of three-dimensional distances from vesicles to active zone for nine fully reconstructed synaptic terminals. Line and shading indicate data fits and 95% confidence intervals. (F) Three-dimensional reconstructions illustrating spatial organization of functional vesicles for two synaptic terminals. Scale bar represents 100 nm. (G) Bar charts comparing the fraction of PC+ vesicles in the nondocked and docked pools (see cartoon). (H) Spatial frequency distribution plot for nonrecycling vesicles (top) and recycling vesicles (bottom) with respect to the center of the active zone generated from normalized projections of 24 synaptic terminals. Scale bars represent 0.1 of normalized distances.

Next, we used our ultrastructural readout of the functional vesicle pool to investigate the spatial organization of recycling vesicles within the presynaptic terminal (Figures 4A and 4B). First, we examined how recycling vesicles were mixed within the total vesicle pool by performing a cluster analysis (n = 368 photoconverted vesicles from 31 synapses). Calculating the recycling fraction in the population of vesicles surrounding each PC+ vesicle at increasing distances from the vesicle center (Figure 4C, inset) showed that at a 50–70 nm radius, the recycling fraction was not significantly different from the baseline fraction for the whole synapse (p values > 0.09, two-tailed one-sample t tests, n = 31), demonstrating that recycling vesicles did not cluster at small distances (Figure 4C). However, a significant peak in the recycling fraction was seen at a 90–110 nm radius (p = 0.02, 0.04, two-tailed one-sample t test, n = 31), after which the fraction tends toward baseline levels as the distance radius approaches the total synapse size (all distances > 110 nm, p values = 0.06–0.98, two-tailed one-sample t tests, n = 31). This demonstrates that recycling vesicles tend to occupy a subset of the total pool area, suggesting a potential spatial bias in vesicle organization (see Figures 4A and 4B). To examine this directly, we analyzed representative middle sections of 24 synaptic terminals and measured the distance from each vesicle—both recycling and nonrecycling—to the nearest point on the active zone and generated cumulative frequency distance plots. These revealed that the distributions of the two populations were significantly different (p < 0.0001, two-tailed paired t test, n = 24), with recycling vesicles occupying positions closer to the active zone than nonrecycling vesicles (Figure 4D). Comparable findings were made for synapses labeled with 4 Hz loading (Figure S2). For the 10 Hz data, we also performed the same analysis on nine fully reconstructed synaptic terminals, which took into account the three-dimensional distance relationships, and this revealed the same preferential bias for recycling vesicles to be close to the release site (Figures 4E and 4F, p < 0.0001, two-tailed paired t test, n = 9).

To test whether this preferential proximity is reflected in the fraction of recycling vesicles in the docked vesicle pool, we next analyzed the composition of docked vesicles and compared it to the composition of the undocked vesicle pool. We found that the proportion of recycling docked vesicles was 0.29 ± 0.04, significantly larger than the fraction of recycling vesicles in the total pool of nondocked vesicles (0.12 ± 0.01, p < 0.01, two-tailed paired t test, n = 41 synapses, Figure 4G). This demonstrates that the tendency for recycling vesicles to be distributed at sites near the active zone is reflected in a larger occupation of the release site itself. Synapses labeled with the 4 Hz loading protocol yielded a comparable result (Figure S2). To analyze our findings further, we measured the position of all vesicles—recycling and nonrecycling—with respect to the center of the active zone and generated a spatial frequency distribution map for each vesicle class, which allowed us to visualize the net organization of the two vesicle pools for 24 synapses. As shown in Figure 4H, the spatial arrangement of the two pools is strikingly different. The nonrecycling pool is broadly distributed around the center of the vesicle cluster but the frequency peak of the recycling pool is biased toward the active zone center and more tightly distributed. These differences in spatial distributions are highly significant (p < 0.0001, two-tailed one-sample t test, n = 24, see Experimental Procedures). Taken together, our findings demonstrate a clear spatial segregation of functional vesicle pools in native presynaptic terminals.

### Regulation of Size of Recycling Pool Fraction by Calcineurin/CDK5 Pathway

**Figure 5:**
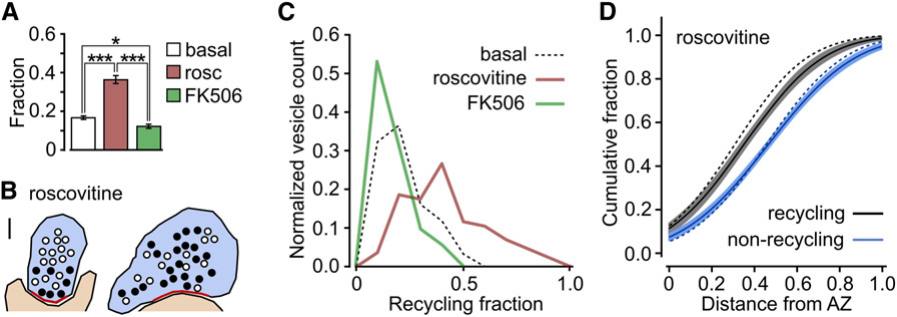
Size of Recycling Pool Fraction Is Regulated by CDK5/Calcineurin Activity (A) Bar chart showing mean ± SEM recycling pool fractions for basal, roscovitine-treated, and FK506-treated synapses. Asterisks indicate significance (^∗^p < 0.05, ^∗∗∗^p < 0.001) for Dunn’s multiple comparison tests (see Results). (B) Sample cartoons illustrating the organization of recycling vesicles (black circles) and nonrecycling vesicles (open circles) in typical roscovitine-treated synapses. Active zones are shown in red. Scale bar represents 100 nm. (C) Frequency distribution for recycling pool fraction for synapses treated with roscovitine (red, n = 86) or FK506 (green, n = 72). Synapses for basal condition are shown with dashed line. (D) Summary cumulative frequency plot of linear distances from vesicles to active zone for recycling and nonrecycling vesicles for 15 central synaptic sections from roscovitine-treated samples. Line and shading indicate data fits and 95% confidence intervals. Dashed lines show data fits for synapses under basal conditions.

The variable nature of the recycling pool fraction seen across populations of synapses suggests that it may be actively regulated under local control. Recent evidence in cultured neurons indicates that the balance of calcineurin and CDK5 activity determines functional pool size (Kim and Ryan, 2010). To test this idea in native synapses, we incubated slices with FK506, a calcineurin inhibitor (Kumashiro et al., 2005; Leitz and Kavalali, 2011), or roscovitine, a CDK5 inhibitor (Kim and Ryan, 2010), before and during synaptic dye labeling. Subsequently, target regions were fixed, photoconverted, embedded, and viewed in ultrastructure. Strikingly, FK506 treatment yielded a significant reduction in the fraction of functional vesicles compared to our basal condition, while roscovitine produced a significant increase (FK506: 0.12 ± 0.01, n = 72; roscovitine: 0.36 ± 0.02, n = 86; basal: 0.17 ± 0.01, n = 93; Kruskal-Wallis test, p < 0.0001, Dunn’s multiple comparison test: FK506 versus basal, p < 0.05; roscovitine versus basal, p < 0.001; FK506 versus roscovitine, p < 0.001) (Figures 5A–5C), consistent with previous findings (Kim and Ryan, 2010; Kumashiro et al., 2005). In some individual synapses from roscovitine-treated slices, the functional pool fraction exceeded 0.8, implying that the majority of vesicles could be converted to recycling ones. Nonetheless, in spite of the roscovitine-driven increase in recycling pool fraction, the preferential spatial organization of recycling vesicles was preserved (p = 0.008, two-tailed paired t test, n = 15, Figure 5D). Thus, an increase in the recycling pool fraction through CDK5 inhibition does not affect the spatial bias of recycling vesicles toward the active zone.

### Disruption of Recycling Pool Segregation by Stabilization of Actin

**Figure 6:**
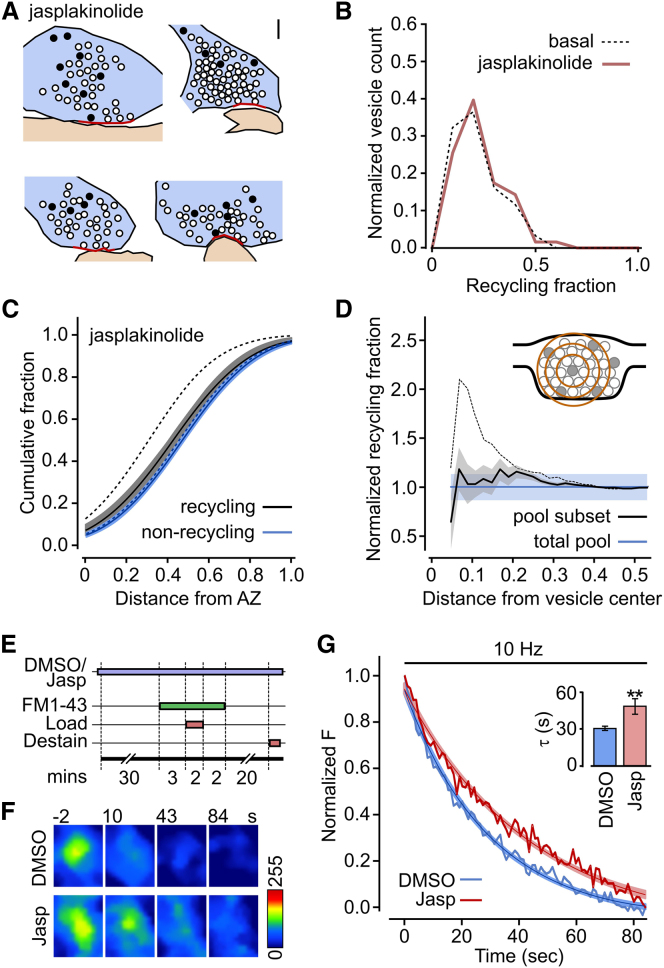
Disruption of Preferential Segregation of the Recycling Pool and Activity-Evoked Fusion Kinetics after Actin Stabilization (A) Sample cartoons illustrating the organization of recycling vesicles (black circles) and nonrecycling vesicles (open circles) in typical jasplakinolide-treated synapses. Scale bars represent 100 nm. (B) Frequency distribution for recycling pool fraction for 63 synapses treated with jasplakinolide (red). Synapses for basal condition are shown with dashed line. (C) Summary cumulative frequency plot of linear distances from vesicles to active zone for recycling and nonrecycling vesicles from 17 central synaptic sections. Line and shading indicate data fits and 95% confidence intervals. Dashed lines show data fits for synapses under basal conditions. (D) Cluster analysis plot showing the mean recycling vesicle fraction for the vesicle population surrounding PC+ vesicles with increasing distance from the vesicle center (see inset). Jasplakinolide abolishes the peak associated with local vesicle clustering seen under basal conditions (dashed line). (E) Schematic of experimental protocol for testing effect of jasplakinolide on vesicle fusion kinetics. (F) Sample images showing activity-evoked FM1-43 dye loss in typical vehicle (DMSO) and jasplakinolide-treated synapses. (G) Average destaining plots with single exponential fits and 95% confidence intervals for DMSO (n = 40) and jasplakinolide-treated synapses (n = 20). Inset: bar chart showing mean ± SEM timecourse for destaining curves. ^∗∗^p = 0.01.

What components might contribute to the preferential organization of recycling vesicles near the active zone? A potential candidate is actin, the highly dynamic cytoskeletal element that is concentrated at synapses (Bloom et al., 2003; Colicos et al., 2001; Sankaranarayanan et al., 2003; Siksou et al., 2011). We tested its possible involvement by incubating slices in the actin-stabilizing agent jasplakinolide before and during synaptic labeling. As with synapses under basal conditions, the average fraction of recycling vesicles in jasplakinolide-treated synapses was small (0.18 ± 0.01, n = 63, Figures 6A and 6B) and similarly distributed (p = 0.32, two-tailed Mann-Whitney test, Figure 6B). Thus, actin does not have a significant role in determining the proportion of recycling vesicles available for turnover. Next, we examined its potential impact on vesicle spatial organization by generating cumulative frequency distance plots. Strikingly, the preferential distribution of recycling vesicles toward the active zone was abolished; both the recycling and nonrecycling pools showed a similar distribution profile (p = 0.38, two-tailed paired t test, n = 17), comparable with the nonrecycling pool profile observed in basal conditions (Figure 6C). We examined how recycling vesicles were mixed with respect to nonrecycling vesicles by performing a cluster analysis. This revealed a flat profile (Figure 6D) with a clear absence of the sharp peak seen under basal conditions and was consistent with a homogeneous mixing of the two pools within the synapse. Taken together, our findings suggest that the impairment of actin remodeling during exo-endocytic vesicle turnover disrupts the overall spatial segregation of recycling vesicles.

The selective effect of jasplakinolide treatment in disrupting spatial segregation allowed us to test for a possible impact of vesicle organization on release properties. Slices were incubated in jasplakinolide or vehicle and subsequently FM dye labeled and destained (Figure 6E) so that we could explore the effects of disrupting the positioning of vesicles on exocytotic kinetics. Fluorescent puncta underwent effective activity-evoked dye loss in both conditions (Figure 6F) but the destaining timecourse was significantly slower in jasplakinolide-treated synapses (p = 0.003, two-tailed Mann-Whitney test, Figure 6G). Although we cannot definitively rule out other possible direct effects of actin disruption on vesicle turnover, our findings provide evidence that the preferential spatial segregation of recycling vesicles serves to increase the efficacy of fast sustained neurotransmitter release.

### Preferential Distribution of Recycling Vesicles Recruited by Sensory Stimulation In Vivo

**Figure 7:**
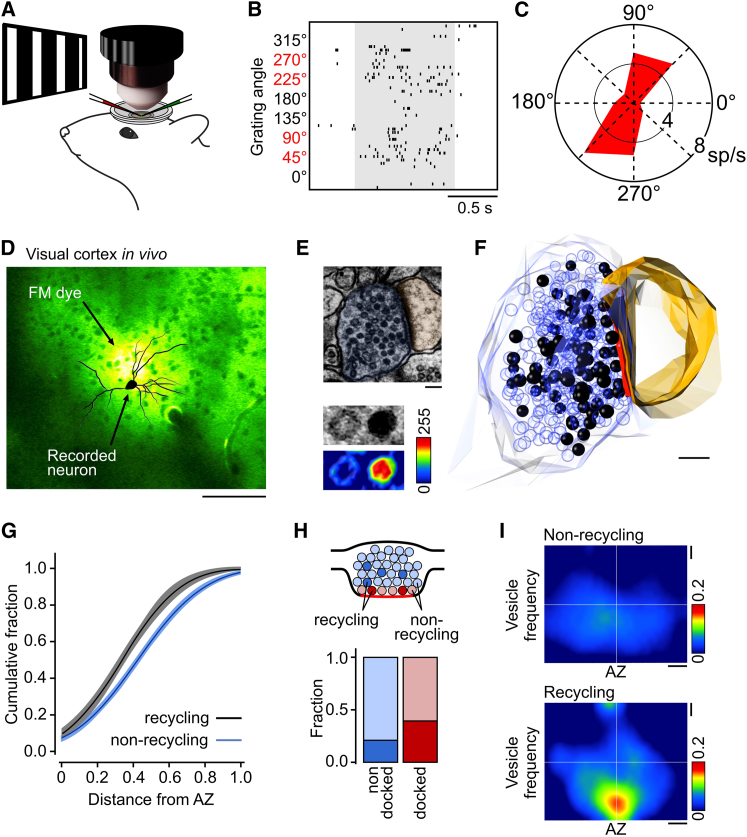
Preferential Segregation of Recycling Vesicles Labeled In Vivo during Visual Processing (A) Schematic of experimental approach: lightly anesthetized mouse viewed visual stimuli (drifting gratings) during whole-cell patch-clamp recording (red pipette) in visual cortex to confirm network activity. Subsequently, FM1-43 dye was applied to region of recorded neuron while repetitive visual stimulation was presented (either a single orientation of a drifting grating or gray screen control). (B) Spike raster plot showing orientation-tuned spike responses (eight directions, six trials/orientation). Gray shading indicates period of visual stimulation. (C) Polar plot showing spike tuning for the same cell as in (B). (D) Two-photon image of visual cortex in vivo showing FM dye fluorescence in region of recorded neuron (reconstruction overlaid). Scale bar represents 100 μm. (E) Top: electron micrograph of synapse with PC+ vesicles. Scale bar represents 100 nm. Bottom: grayscale and intensity plot with pseudocolor look-up table illustrates different lumenal density profiles for PC+ (red lumen) and PC− (blue lumen) vesicles. (F) Full three-dimensional reconstruction of synapse in (E) based on 12 consecutive serial sections. Scale bar represents 100 nm. (G) Summary cumulative frequency plot of linear distances from vesicles to active zone for recycling and nonrecycling vesicles from 17 central synaptic sections. Line and shading indicate data fits and 95% confidence intervals. (H) Bar charts comparing the fraction of PC+ vesicles in the nondocked and docked pools (see cartoon). (I) Spatial frequency distribution plot for nonrecycling vesicles (top) and recycling vesicles (bottom) with respect to the center of the active zone generated from normalized projections of 17 synaptic terminals. Scale bars represent 0.1 of normalized distances.

How do our findings in acute slice relate to function-structure properties of central synapses in intact brain? To address this question, we used the same FM dye loading, photoconversion, and ultrastructure approach, but this time in visual cortex in vivo where vesicle recycling could be driven by the presentation of a defined sensory stimulus (Figure 7A). First, we performed in vivo whole-cell recordings during presentation of defined visual stimuli (drifting square-wave gratings) to confirm network activity (Figures 7A and 7B) and revealed robust orientation-tuned spike responses (Figures 7B and 7C). Next, FM1-43 was applied to the recording region (Figure 7D) while repetitive visual stimulation (10 min) was presented to drive vesicle recycling. The animal was then sacrificed and the brain fixed, sliced, photoconverted, and prepared for ultrastructural analysis. In electron micrographs from the target region, activated synapses were evidenced by PC+ vesicles (Figures 7E and 7F), analogous to those seen in our hippocampal experiments. As expected, in control synapses from mice presented with a gray screen visual stimulus during dye labeling, the average fraction of PC+ vesicles was significantly lower (gray screen: 0.03 ± 0.01, n = 30; grating: 0.13 ± 0.02, n = 35; based on randomly collected samples for each condition; p = 0.0002, Mann-Whitney t test; Figure S3). Next, we examined the spatial organization of functionally recycling vesicles by generating cumulative frequency distance plots for activated synapses (average recycling fraction: 0.23 ± 0.04, n = 17). Notably, there was a preferential spatial organization of recycling vesicles toward the active zone (p = 0.008, two-tailed paired t test, n = 17, Figure 7G) and a larger representation in the docked vesicle pool (Figure 7H), analogous to our findings in hippocampus. Furthermore, spatial frequency distribution maps for the two vesicle classes matched our previous results, showing that the spatial arrangement of the two pools was different with the frequency peak of the recycling pool biased toward the active zone center and more tightly distributed (p < 0.0001, two-tailed one-sample t test, n = 17, Figure 7I). Taken together, our findings extend the observation of a spatially segregated functional vesicle pool to presynaptic terminals in vivo.

## Discussion

Here we combined FM dye labeling with photoconversion and serial electron microscopy to examine the ultrastructural organization of the recycling vesicle pool in small native central synapses. This approach provides a selective readout of the functional pool that can be directly related to the morphological ultrastructure of the same synaptic terminals. Our findings offer important insights into the relationship between pool size and synapse size. Additionally, spatial analysis reveals shared features of vesicle organization in different types of small central synapse, suggesting that physical positioning of vesicle pools may be an important factor in their favored release.

Our findings provide important insights into structure-function relationships in presynaptic terminals, an issue which has attracted considerable recent interest (Holderith et al., 2012; Sheng et al., 2012). Specifically, we demonstrate that the average functional pool size in synapses from acute hippocampal slices is small (approximately one-fifth of the total pool). From work in cultured hippocampal neurons, there is no clear consensus on the magnitude of the recycling pool fraction, with a wide range of reported values (Branco et al., 2010; Darcy et al., 2006a; Fernandez-Alfonso and Ryan, 2008; Fredj and Burrone, 2009; Ikeda and Bekkers, 2009; Kim and Ryan, 2010; Li et al., 2005; Micheva and Smith, 2005; Welzel et al., 2011), although some are directly comparable with our findings (∼15%–20%) (Harata et al., 2001a, 2001b). In studies from other native terminals, recycling fractions can also be relatively small (de Lange et al., 2003; Rizzoli and Betz, 2004) and recent work demonstrates that a very small functional pool (1%–5%) is sufficient to support naturally driven or spontaneous vesicle turnover in a range of native, mostly peripheral terminals (Denker et al., 2011). Taken together, this suggests that, across a range of synapse types from native tissue, a limited subset of the total vesicle pool is typically used during synaptic transmission. While we found a broad scaling of the recycling pool size with other parameters of synaptic morphology (see also Harris and Sultan, 1995; Schikorski and Stevens, 1997), the fraction of recycling vesicles was highly variable and not related to total vesicle pool size, suggesting that, at individual terminals, this parameter could be independently regulated. Importantly, recent work in cultured hippocampal neurons has demonstrated that modulation of the recycling pool fraction is associated with forms of activity-dependent plasticity (Kim and Ryan, 2010; Ratnayaka et al., 2012) and that CDK5 and calcineurin are important control points in such regulation (Kim and Ryan, 2010). Our current results provide support for this in native synapses; we show that inhibition of CDK5 activity doubles the average recycling pool fraction, while inhibition of calcineurin reduces it by a third. Taken together, these findings support an emerging view of the recycling fraction as a modifiable parameter contributing to synapse operation; we suggest that its limited average size in native tissue confers a broad dynamic range over which synaptic performance can be adjusted.

Our analysis of the spatial positions occupied by recycling vesicles within the total vesicle cluster in native hippocampal synapses reveals a strong preferential bias toward sites nearer to the active zone. Importantly, recycling vesicles were not significantly clustered at short distances but instead were distributed within a subset of the total cluster volume. Moreover, recycling vesicles were not confined just to sites that were close to the active zone; the fraction of photoconverted vesicles within the docked vesicle pool was much higher than expected by chance. This suggests a model in which recently recycled vesicles take up positions, on average, toward the front of the vesicle cluster. This finding is broadly consistent with a landmark study in dissociated hippocampal cultured neurons looking at a different functional pool—the readily releasable pool—which characterized the tendency for vesicles to occupy positions close to the active zone (Schikorski and Stevens, 2001). In theory, our total recycling pool could include a subset of preferentially reused vesicles (Ertunc et al., 2007; Pyle et al., 2000) and the spatial bias we observe here could be indicative of a fast mode of recycling (Gandhi and Stevens, 2003; Park et al., 2012; Zhang et al., 2009); further work will be needed to test the relevance of these ideas in native terminals.

To explore the generality of our findings, we also used a modified form of our FM dye photoconversion method to characterize the nanoscale appearance of functional vesicle pools in vivo, in this case, specifically recruited by activity driven by defined sensory input. This report establishes an experimental strategy for delineating function-ultrastructure characteristics of synapses from intact brain. Notably, our findings regarding functional pool organization in visual cortex were highly consistent with those in hippocampal slices: functional vesicles were preferentially located near the active zone, suggesting that this is a shared feature among different types of small central synapses.

We investigated a possible role for the cytoskeletal element actin as a candidate in contributing to spatial segregation. We showed that stabilizing actin with jasplakinolide disrupted the preferential distribution of recycling vesicles, indicating that remodeling actin is important in facilitating the repositioning of recycling vesicles toward the active zone after endocytosis. These findings are broadly compatible with the current model for actin function in the presynaptic terminal as a scaffolding element, guiding vesicle-associated components to their destination during repeated cycles of activity (Sankaranarayanan et al., 2003; Shupliakov et al., 2002) (also see Pechstein and Shupliakov, 2010). Importantly, we show that actin stabilization, and by association the abolition of preferential recycling pool distribution, does not prevent vesicle turnover but does affect the rate of release; experiments measuring FM dye loss show clear stimulation-evoked destaining but notably the timecourse of exocytosis is significantly slower compared to controls. Given that a clear direct role for actin in driving synaptic vesicle exocytosis has not been established (Sankaranarayanan et al., 2003), the effects we observe most likely result from disruption of the recycling pool distribution. We suggest that the preferential spatial positioning of functional vesicles might contribute to efficient vesicle release during sustained activity.

Interestingly, the segregation of recycling vesicles toward release sites is not a universal property of presynaptic terminals. Studies on neuromuscular junctions (Denker et al., 2011; Rizzoli and Betz, 2004) and mammalian calyx of Held terminals (de Lange et al., 2003) have previously demonstrated that releasable vesicles are not preferentially arranged but mixed randomly in the total vesicle pool. In the case of the frog neuromuscular junction, this is particularly significant because the ultrastructurally labeled vesicles, corresponding to the readily releasable pool, are likely to undergo preferential reuse (Richards et al., 2000, 2003; Rizzoli and Betz, 2004). This implies that their privileged status must be conferred by factors other than their specific spatial relationship to the active zone. A plausible hypothesis is that these vesicles might retain only loose coupling with the vesicle cluster and could have preferential access to the release site by way of cytoskeletal tracks that link them to the active zone (Rizzoli and Betz, 2004). Our findings indicate that the total recycling pool in hippocampal synapses is also preferentially reused, but in the case of these size-limited terminals, vesicle positioning appears to be an important parameter in conferring privileged release. Interestingly, recent work has shown conclusively that different functional vesicle classes have different molecular signatures (Hua et al., 2011), providing a possible mechanistic basis for the selective regulation and distribution of the functional vesicle pool subsets that we have demonstrated here.

## Experimental Procedures

### Acute Slice Preparation

Experiments were performed in accordance with the UK-Animal (Scientific Procedures) Act 1986 and complied with local institutional regulations. Acute transverse slices of hippocampus (300 μm) were prepared from 3- to 4-week-old rats and maintained in artificial cerebrospinal fluid (aCSF) containing 125 mM NaCl, 2.5 mM KCl, 25 mM glucose, 1.25 mM NaH_2_PO_4_, 26 mM NaHCO_3_, 1 mM MgCl_2_, 2 mM CaCl_2_, 20 mM μM CNQX, and 50 mM μM AP5 (pH 7.3 when bubbled with 95% O_2_ and 5% CO_2_) (see also Ratnayaka et al., 2011; Staras et al., 2010; Zakharenko et al., 2001). Live labeling of functional presynaptic terminals used FM1-43FX, the fixable form of the styryl dye (Molecular Probes). We pressure applied 20 μM FM1-43FX in aCSF to the CA1 region for 3 min prior to stimulation. Schaffer collaterals were stimulated using a bipolar tungsten electrode (Figure 1A). FM1-43 solution was puffed throughout the stimulation period and for 2 min after the end of stimulation to ensure full completion of endocytosis (Granseth and Lagnado, 2008). Subsequently, slices were perfused continuously in fresh aCSF for 15–20 min at 25°C to wash residual FM dye from extracellular membranes. The imaging of FM dye-labeled presynaptic terminals was performed using an Olympus BX51WI microscope equipped with an FV-300 confocal system (Olympus UK), a 488 nm Argon laser, and 520/10 emission. When indicated, slices were continuously perfused with 100 μm roscovitine (Calbiochem) or 1 μm FK506 (Tocris) or jasplakinolide (Calbiochem) in aCSF starting 30 min prior to FM dye loading; for DMSO-control experiments, 1 μl/ml DMSO was used.

### Photoconversion and Electron Microscopy Preparation

FM1-43FX-loaded slices were fixed using rapid microwave fixation in 6% gluteraldehyde, 2% formaldeahyde in PBS as described previously (Jensen and Harris, 1989). After fixation, the samples were transferred into 100 mM glycine in PBS (1 hr), then rinsed in 100 mM ammonium chloride (1 min) and washed in PBS. For photoconversion, the slices were incubated in an oxygen-bubbled diaminobenzidine solution (DAB, 1 mg/ml, Kem En Tec diagnostics). The DAB solution was refreshed after 10 min and the region of interest was illuminated with intense blue light (<500 nm from a Mercury lamp) for 22–25 min. After photoconversion, the samples were prepared for electron microscopy using an established protocol (Jensen and Harris, 1989). Briefly, the samples were placed in 1% osmium tetroxide (Agar Scientific) and 1.5% potassium ferrocyanide (Sigma) in cacodylate buffer and, after osmication, stained en block in uranyl acetate and dehydrated for embedding in EPON resin (TAAB). Sectioned samples were laid on bare mesh or formvar-coated slot grids and sections collected between ∼5 and ∼15 μm from the photoilluminated surface (see also Figure S1) were viewed using a Hitachi-7100 transmission electron microscope. Digital images were acquired using a 2,048 × 2,048 charge-coupled device camera (Gatan).

### In Vivo Surgeries

Wild-type C57/blk6 mice (24–56 days old) were anesthetized with isoflurane (5% for induction, 1.5%–2.5% for surgery, and 0%–0.5% during recording), augmented with chlorprothixene (0.5–2 mg/kg, intraperitoneally). A 2–3 mm diameter craniotomy was opened over visual cortex. The dura mater was left intact. A thin layer of agar (1.5%) dissolved in aCSF (150 mM NaCl, 2.5 mM KCl, 10 mM HEPES, 2 mM CaCl_2_, and 1 mM MgCl_2_; pH adjusted with NaOH to 7.3; 300 mOsm) and placed on top of the brain helped dampen movement. A homeothermic heat pad maintained body temperature within the physiological range. Water-based opthalmic ointment maintained eye health.

### Visual Stimulus Presentation

Visual stimulus presentation was controlled by routines written in MATLAB using the Psychophysics Toolbox extensions (Brainard, 1997; Kleiner et al., 2007, Perception 36 ECVP, abstract; Pelli, 1997). Square-wave gratings (0.04 cycles/deg, 2 cycles/s) of black (2 cd/m2) and white (86 cd/m2) bars in eight different orientations were displayed on an LCD screen (ESAW 7 inch VGA TFT, set at 1,024 × 768 resolution and 60 Hz refresh rate) to map orientation selectivity. For control gray screen stimulation, the total luminance was matched to that of the grating stimulus. The screen was shrouded with a cone up to the eye of the mouse to prevent contamination of the imaging pathway with light from the visual stimulus. The visual stimulus extended from +20° to +124° in azimuth and from −10° to +42° in elevation.

### In Vivo Two-Photon-Guided Electrophysiology and FM Dye Bulk Loading

A custom-built two-photon microscope using galvanometer-based scan mirrors (6 mm diameter, Cambridge Technologies) with a 16× magnification and 0.8 numerical aperture water-immersion objective (Nikon) and a large aperture collection pathway with low-noise photomultiplier tubes (models 3896 and 7422-40P, Hamamatsu) was used to image neurons. The software ScanImage (Pologruto et al., 2003) was used to control the microscope.

For somatic patch-clamp recordings, the pipette solution contained 135 mM KMeSO4, 4 or 10 mM KCl, 10 mM HEPES, 10 mM Na_2_-phosphocreatine, 4 mM Mg-ATP, 2 mM Na_2_-ATP, 0.3 mM Na_2_-GTP, 0.1 mM Oregon green BAPTA-1, and 0.025–0.050 mM Alexa 594; pH adjusted with KOH to 7.2; 290 mOsm. Pipette resistances ranged from 5 to 8 MΩ. Shadowpatching techniques (Kitamura et al., 2008) were used to directly target the pipette to the soma. Series resistance was 39 ± 5 MΩ.

For in vivo labeling of functional recycling synaptic vesicles at the site of electrophysiological recordings, FM1-43FX was bolus loaded into neurons. Under two-photon microscopy, a patch pipette containing 20 μM FM1-43FX in aCSF was guided in the vicinity of a previously patched and fluorescently labeled pyramidal neuron. Pressure of 300–600 mbar was applied for 1–3 min to eject FM dye solution from the pipette. This stained a spherical volume of 300–400 μm in diameter. After visual stimulation, the animal was anaesthetized with ketamine/xylazine and perfusion fixed via cardiac injection with 4% gluteraldehyde, 4% paraformaldeahyde (average time between visual stimulation and end of fixation was ∼10 min). The brain was removed, 100 μm coronal slices were prepared, and the slice containing the region of interest was then photoconverted.

### Analysis

Confocal images and electron micrographs were analyzed using ImageJ (NIH). Destaining analysis was performed with regions of interest that encapsulated synaptic puncta. At ultrastructural level, target synapses were randomly chosen and synaptic vesicles were scored as photoconverted (PC+) or nonphotoconverted (PC−) based on their vesicle lumenal intensity using methods outlined previously (Darcy et al., 2006a, 2006b). Vesicles were sometimes observed in axons consistent with previous findings (Shepherd and Harris, 1998); to ensure that we were analyzing the synaptic vesicle cluster, we defined its boundary as the point where vesicles were separated by 200 nm in a line running away from the active zone center. Synapses outside the photoconversion region did not have any PC+ vesicles (Figure S1). Synapses in photoconverted regions that were incubated in FM dye but not stimulated occasionally contained PC+ vesicles (mean fraction: 0.005, corresponding to 11 positive vesicles from 92 synapses analyzed), presumably a result of spontaneous and nonstimulus-specific release. To ensure that this stimulus-independent labeling was not included in our data set, we set a lower threshold for inclusion in the data set based on this mean fraction +2 × SD (see Figure S1). Micrographs were aligned and reconstructed using Xara Xtreme and Reconstruct (Synapse Web, Kristen M. Harris, http://synapses.clm.utexas.edu). Shapiro-Wilk normality test was performed on all data sets to determine the appropriate type of statistical analysis to carry out. ANOVA was used to examine variation across multiple groups with post hoc Dunn’s multiple comparison tests. Two-tailed Spearman’s test was used to compare correlations. One-sample and paired t tests were used for comparisons of clustering, distribution, and docking. To compare the total spatial distribution of PC+ versus PC− vesicles (Figure 4H), we computed the difference between the spatial frequency histograms. This was done on a bin-by-bin basis for the bins with the highest 70% frequencies of the PC+ cluster (i.e., the spatial area encompassing 70% of PC+ vesicles). The distribution of differences was then tested with a one-sample t test under the null hypothesis that the mean difference was 0. The alpha value of 0.05 was used for all statistical comparisons.

### Modeling

To investigate the effect of preferential reuse of recycling vesicles on FM dye destaining curves, we implemented a stochastic model of vesicle release in Python. The model had a recycling pool of 40 vesicles, with a release probability of 0.15 and a recycling time of 10 s. All recycling vesicles were initially labeled as FM positive, and the synapse was stimulated at 10 Hz while monitoring the decrease in the number of FM-positive vesicles. The fraction of reuse was varied between 100% and 0% by drawing vesicles from a pool with the desired fraction of FM-positive and FM-negative vesicles. Statistical comparison between the model and experimental data used a two-sample t test for each time point, and mean alpha value for the whole curve was then calculated. The mean alpha value was >0.05 for reuse fractions between 95% and 80%, and the highest value was for 88% reuse (p = 0.28).
