# Supplementary material for: A Preferentially Segregated Recycling Vesicle Pool of Limited Size Supports Neurotransmission in Native Central Synapses
Source: Neuron. 2012 Nov 8;76(3-3):579–89. doi: 10.1016/j.neuron.2012.08.042 (PMC3526798; doi:10.1016/j.neuron.2012.08.042)
Supplement: Document S1. Figures S1–S3 [file mmc1.pdf]

Neuron, Volume *76*

Supplemental Information

A Preferentially Segregated Recycling Vesicle Pool

of Limited Size Supports Neurotransmission

in Native Central Synapses

Vincenzo Marra, Jemima J. Burden, Julian R. Thorpe, Ikuko T. Smith, Spencer L. Smith, Michael Häusser, Tiago Branco, and Kevin Staras

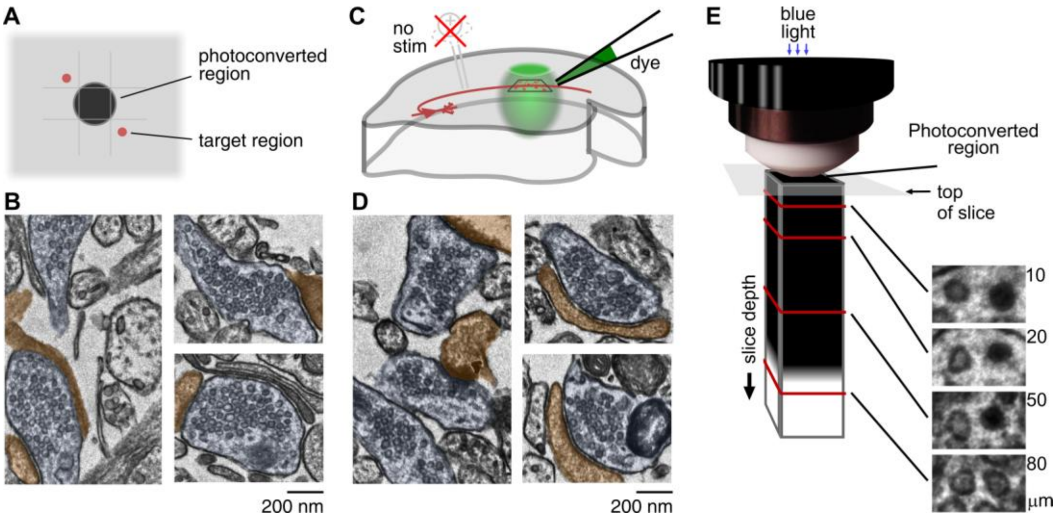

Figure S1. (Related to Figure 2). Photoillumination, dye-labeling and depth controls for photoconversion. (**A**) Schematic illustrating control condition for the light-dependence of DAB oxidation. Cartoon shows the photoconverted region (black circle) viewed from the top of the slice with score marks used to identify area in EM. Small red circles illustrate target regions laterally displaced from the photoconverted site. (**B**) Typical terminals at non-photoconverted sites (eg. red circles from a); no photoconverted vesicles were seen in these regions (n > 500 synapses). (**C**) Schematic illustrating non-stimulation control. Slice labeling protocol was performed with FM-dye present but without stimulation of Schaffer collaterals. DAB photoconversion and processing were carried out as normal. (**D**) Typical terminals for the non-stimulation control condition. We observed a very small number of photoconverted vesicles; an average fraction of 0.005 ± 0.002 from 92 synapses. These presumably arose from spontaneous recycling events in the period when neurons were incubated in FM-dye. (**E**) Effectiveness of the photoconversion reaction with increasing tissue depth. We targeted photoconverted regions and collected sample sections at increasing depths through the slice (left cartoon). Synapses with photoconverted vesicles could be readily observed from 2 to 50 μm below the slice surface (right panel). No photoconverted vesicles were observed at 80 μm or below (right panel), suggesting insufficient light penetration to drive photo-oxidation at this depth. All synapses used for analysis in the paper were taken from sections in a depth range of 5 - 15 μm.

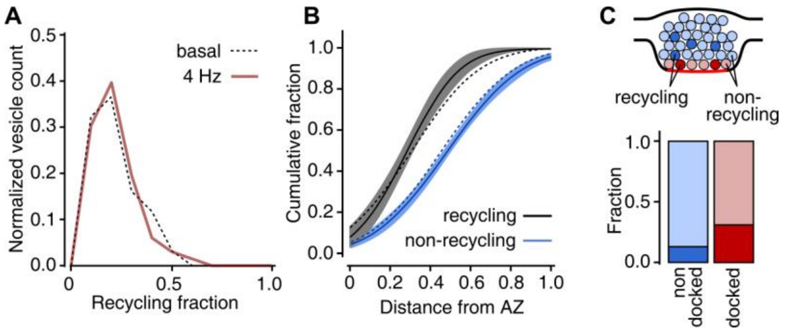

Figure S2. (Related to Figure 4). Properties of vesicle pools labeled with 4 Hz 1200 AP loading protocol. (**A**) Frequency distribution for recycling pool fraction for 68 synapses loaded at 4 Hz. Synapses for basal condition are shown with dashed line (P = 0.52, two-tailed Mann-Whitney test). (**B**) Summary cumulative frequency plot of linear distances from vesicles to active zone for recycling and non-recycling vesicles from 17 central synaptic sections. Line and shading indicate data fits and 95% confidence intervals (P = 0.02, two-tailed paired t-test, n = 7). Dashed lines show data fits for synapses under basal conditions. (**C**) Bar charts comparing the fraction of PC+ vesicles in the non-docked and docked pools (see cartoon).

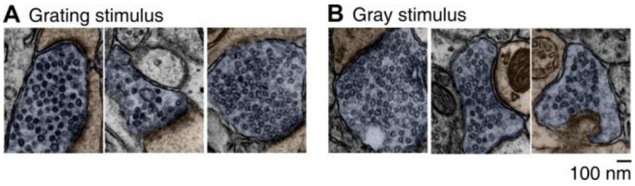

Figure S3. (Related to Figure 7). Stimulus-dependent labeling of synaptic vesicles in visual cortex. Representative images from synapses loaded with FM-dye during presentation of a grating stimulus (A) or gray stimulus (B). The fraction of photoconverted vesicles in randomly sampled synapses for each condition was significantly different (grating: 0.13 ± 0.02, gray: 0.03 ± 0.01, P = 0.0002, two-tailed unpaired t-test, n = 35 and n = 30).
